# Socio-ecological predictors of mental health outcomes among healthcare workers during the COVID-19 pandemic in the United States

**DOI:** 10.1371/journal.pone.0246602

**Published:** 2021-02-05

**Authors:** Rachel Hennein, Emma J. Mew, Sarah R. Lowe

**Affiliations:** 1 Yale School of Medicine, Yale University, New Haven, Connecticut, United States of America; 2 Department of Epidemiology of Microbial Diseases, Yale School of Public Health, Yale University, New Haven, Connecticut, United States of America; 3 Department of Chronic Disease Epidemiology, Yale School of Public Health, Yale University, New Haven, Connecticut, United States of America; 4 Department of Social and Behavioral Sciences, Yale School of Public Health, Yale University, New Haven, Connecticut, United States of America; University of the Witwatersrand, SOUTH AFRICA

## Abstract

**Background:**

Healthcare workers are at increased risk of adverse mental health outcomes during the COVID-19 pandemic. Studies are warranted that examine socio-ecological factors associated with these outcomes to inform interventions that support healthcare workers during future disease outbreaks.

**Methods:**

We conducted an online cross-sectional study of healthcare workers during May 2020 to assess the socio-ecological predictors of mental health outcomes during the COVID-19 pandemic. We assessed factors at four socio-ecological levels: individual (e.g., gender), interpersonal (e.g., social support), institutional (e.g., personal protective equipment availability), and community (e.g., healthcare worker stigma). The Personal Health Questionnaire-9, Generalized Anxiety Disorder-7, Primary Care Post-Traumatic Stress Disorder, and Alcohol Use Disorders Identification Test-Concise scales assessed probable major depression (MD), generalized anxiety disorder (GAD), posttraumatic stress disorder (PTSD), and alcohol use disorder (AUD), respectively. Multivariable logistic regression models were used to assess unadjusted and adjusted associations between socio-ecological factors and mental health outcomes.

**Results:**

Of the 1,092 participants, 72.0% were female, 51.9% were frontline workers, and the mean age was 40.4 years (standard deviation = 11.5). Based on cut-off scores, 13.9%, 15.6%, 22.8%, and 42.8% had probable MD, GAD, PTSD, and AUD, respectively. In the multivariable adjusted models, needing more social support was associated with significantly higher odds of probable MD, GAD, PTSD, and AUD. The significance of other factors varied across the outcomes. For example, at the individual level, female gender was associated with probable PTSD. At the institutional level, lower team cohesion was associated with probable PTSD, and difficulty following hospital policies with probable MD. At the community level, higher healthcare worker stigma was associated with probable PTSD and AUD, decreased satisfaction with the national government response with probable GAD, and higher media exposure with probable GAD and PTSD.

**Conclusions:**

These findings can inform targeted interventions that promote healthcare workers’ psychological resilience during disease outbreaks.

## Introduction

Since the onset of the coronavirus disease 2019 (COVID-19) pandemic, over 21 million people in the United States (US) have tested positive and over 360,000 have died, making the US the most impacted country worldwide [1]. The rapid spread of COVID-19 across the US has put significant strain on healthcare workers (HCWs) directly and indirectly combatting the pandemic, which could increase risk of adverse mental health outcomes. Indeed, a meta-analysis of mental health outcomes during the first wave of the COVID-19 pandemic identified a 23.2% prevalence of anxiety and 22.8% prevalence of depression among HCWs [2]. This meta-analysis notably did not include any study of HCWs in the US, however.

Another rapid review including studies from the COVID-19 and other pandemics up to August 21, 2020 also concluded that HCWs are at increased risk for symptoms of posttraumatic stress disorder (PTSD), major depression (MD), and generalized anxiety disorder (GAD) [3]. Of the 38 articles focused on the COVID-19 pandemic, only one was conducted in the US, using a sample of 657 HCWs in New York City (NYC) during the first wave of the pandemic [4]. The authors found that 57% of the respondents screened positive for PTSD symptoms, 48% for MD symptoms, and 33% for GAD symptoms. Additional studies are warranted in other geographic locations throughout the US to quantify the mental health impacts of the pandemic on HCWs.

It is likely that mental health outcomes among HCWs are related to risk and protective factors at various socio-ecological levels, including *individual* (e.g., age, gender, and occupation), *interpersonal* (e.g., social support), *institutional* (e.g., personal protective equipment [PPE] availability), and *community* (e.g., stigma) factors [5–7]. Studies to date have provided evidence for the importance of factors at each level. For example, female gender in China and Italy [8–10], nurse occupation in China and the US [3, 4, 8], frontline status in China, Italy, and the US [8–11], decreased social support in the US and China [4, 9, 12, 13], low PPE availability in Iran [14], and HCW stigma in China [9] have been associated with psychological distress among HCWs during the COVID-19 pandemic. Most of these studies took place in China, and none to our knowledge simultaneously examined factors at all four socio-ecological levels. Additional studies are therefore needed to understand the range of socio-ecological factors contributing to mental health outcomes among HCWs in the US.

To fill this gap, we surveyed HCWs from 25 academic medical centers across the US during the COVID-19 pandemic to assess socio-ecological factors associated with four mental health outcomes: MD, GAD, PTSD, and alcohol use disorder (AUD). By conducting multivariable logistic regression models that included factors from four different socio-ecological levels (i.e. individual, interpersonal, institutional, and community), we sought to identify significant predictors of these outcomes among HCWs. Our findings can provide important insights for interventions to support HCWs during the COVID-19 pandemic and future outbreaks.

## Methods

### Setting

The methods of this cross-sectional study are described elsewhere [15]. Briefly, we distributed an online survey to HCWs affiliated with 25 medical centers across the US for the entirety of May 2020. The survey was launched one week after the first peak of documented COVID-19 cases in the US, with almost 2 million cumulative cases and 100,000 deaths [16]. We sampled teaching hospitals in each region of the US, including Northeast, Midwest, South, and West. Within each region, we purposively sampled hospitals in states with high rates of COVID-19 transmission determined by a geographical mapping tool of COVID-19 transmission data [16]. Our study was approved by the Yale Institutional Review Board and all participants provided written consent. This manuscript followed the Strengthening the Reporting of Observational Studies in Epidemiology (STROBE) reporting guideline for cross-sectional studies [17].

### Eligibility and recruitment

We contacted hospital department chairs to invite them to forward our survey to their staff. HCWs at least 18 years of age were eligible for inclusion. Our study was open to physicians, medical trainees, nurses, clinical assistants, health technologists/technicians, and non-clinical personnel. A total of 1132 HCWs participated; of those, 1092 (96.5%) completed all measures in the current study and comprised the analytic sample.

### Data collection tool

#### Mental health measures

We included measures of key mental health outcomes with prior evidence of strong psychometric properties: the Patient Health Questionnaire-9 (PHQ-9) to assess MD symptoms [18, 19]; Generalized Anxiety Disorder-7 (GAD-7) to assess GAD symptoms [20]; Primary Care-PTSD (PC-PTSD) to assess PTSD symptoms [21]; and Alcohol Use Disorders Identification Test-Concise (AUDIT-C) to assess AUD symptoms [22]. Based on validation studies for each measure [18–22], we defined probable depression as PHQ-9> = 10, probable GAD as GAD-7> = 10, probable PTSD as PC-PTSD> = 3, and probable AUD as AUDIT-C> = 4 for men and AUDIT-C> = 3 for women.

#### Socio-ecological factors

We selected socio-ecological factors based on previous studies on mental health outcomes among HCWs during pandemics [8, 23, 24]. These factors can be mapped to four ecological levels: individual, interpersonal, institutional, and community [5–7].

*Individual-level factors*. Individual-level factors included age, gender, ethnicity, race, marital status, pre-pandemic psychiatric illness, geographic region, COVID-19 status, frontline status, and profession. We assessed for COVID-19 status by asking respondents if they have previously tested positive for COVID-19. Frontline status was assessed by asking respondents if they “directly worked in COVID-19 patients’ rooms” (i.e. direct exposure), “worked in COVID-19 patient care remotely only” (i.e. indirect exposure), or “did not work with patients with COVID-19” (i.e. no exposure). Specialty risk levels included low (e.g., dermatology), moderate (e.g., gastroenterology), and high (e.g., critical care) based on studies that calculated percentage of HCWs who tested positive for COVID-19 by specialty [25, 26]. For example, one study calculated the percentage of HCWs who tested positive for COVID-19, stratified by specialty, in a teaching hospital in the United Kingdom [25]. Another study calculated the percentage of resident physicians who tested positive for COVID-19 by specialty in New York City [26]. We rank-ordered these COVID-19 risk statistics by specialty to categorize them as low, moderate, or high risk.

*Interpersonal-level factors*. Interpersonal-level factors included social support, measured using one item from the Social Support Questionnaire from the National Health and Nutrition Examination Survey [27], and a dichotomous indicator of whether participants had experienced a change in their living situation due to COVID-19.

*Institutional-level factors*. Questions to assess institutional-level factors were based on survey items that assessed HCW wellbeing during previous pandemics, including changes in hospital role, changes in work hours, and shortages of PPE [23, 24]. Participants also answered three questions assessing the extent to which hospital/clinic policies to limit nosocomial COVID-19 transmission were transparent, timely, and difficult to follow using 5-point Likert scales. Team cohesion was measured using validated questions from the Survey of Organizational Attributes for Primary Care [28, 29] and novel questions to assess supervisor support, rated using 5-point Likert scales. Specifically, participants rated the extent to which: conflict on their hospital/clinic teams are communicated and resolved, all staff participates in important decisions about clinical operations, their hospital/clinic team has been a source of support to get through the pandemic, the staff members feel overwhelmed by work demands, supervisors are available for consultation, and supervisors acknowledge their work for the team.

*Community-level factors*. Community-level factors included single items, rated on 5-point Likert scales, assessing perceived societal appreciation for HCWs, perceived stigmatization of being a HCW, and satisfaction with local/state and national government responses to COVID-19. For example, perceived HCW stigmatization was ascertained by asking respondents to assess the extent to which the following statement is true: “I am negatively stigmatized because I am a healthcare worker during the COVID-19 pandemic.” Media exposure was assessed by asking the number of hours that respondents spent consuming media coverage of COVID-19 daily.

### Data analysis

Descriptive statistics were computed for all variables in the analysis, and independent-samples *t*-tests and chi-square analysis assessed for differences between participants in the analytic sample and those dropped due to missing data. Next, analyses predicting probable MD, GAD, PTSD, and AUD were conducted. Unadjusted binary logistic regression models assessed bivariate relationships between individual-, interpersonal-, institutional-, and community-level factors and each mental health outcome. Subsequently, all socio-ecological factors were entered simultaneously in adjusted models. Reference groups for categorical variables were selected based on either prior research findings indicating decreased risk or descriptive data indicating symptom levels distinctively lower than other groups. For example, male was selected as the reference group for gender, given a large body of research linking female gender to increased risk for MD, GAD, and PTSD [30, 31]. Midwest was selected as the reference group for geographic region, given that the prevalence estimates for all four mental health outcomes were descriptively lower among participants residing there relative to those residing in all three other regions. Analyses were conducted in SPSS 27.0 (IBM Corp., 2020). We considered a *p*-value of less than 0.05 to be statistically significant.

## Results

### Preliminary analyses

Table 1 shows descriptive statistics for all variables in the analysis. Participants were on average 40.44 years old (*SD* = 11.52). The majority identified as female (72.0%) and white (78.8%), and 64.7% were married. Based on geographic region, 53.9%, 9.4%, 24.2%, and 12.4% of respondents worked in the Northeast, South, Midwest, and West, respectively. Over half (51.9%) reported direct exposure to COVID-19 patients, 17.6% indirect exposure, and 30.5% as no exposure. Based on their symptom inventory scores, 13.9%, 15.6%, 22.8%, and 42.8% were classified as having probable MD, GAD, PTSD, and AUD, respectively. No significant differences were detected between participants in the analytic sample (n = 1092) and those dropped due to missing data (n = 38).

**Table 1:** Descriptive statistics of all variables in the analysis.

* *	Mean	Standard deviation	%	N
**Prevalence of probable mental health conditions**				
Major depression	N/A	N/A	13.9	152
Generalized anxiety disorder	N/A	N/A	15.6	170
Posttraumatic stress disorder	N/A	N/A	22.8	249
Alcohol use disorder	N/A	N/A	42.8	467
**Individual-level factors**				
Age (years)	40.44	11.52	N/A	N/A
Gender				
Female	N/A	N/A	72.0	785
Male	N/A	N/A	28.0	305
Hispanic	N/A	N/A	5.6	61
Race				
White	N/A	N/A	78.8	859
Black or African American	N/A	N/A	4.8	52
Asian ethnicity	N/A	N/A	13.1	143
Other	N/A	N/A	2.3	25
Marital status				
Married	N/A	N/A	64.7	705
Single	N/A	N/A	28.4	310
Divorced or widowed	N/A	N/A	6.9	75
Reported pre-pandemic psychiatric illness	N/A	N/A	24.6	268
Region				
Northeast	N/A	N/A	53.9	588
South	N/A	N/A	9.4	103
Midwest	N/A	N/A	24.2	264
West	N/A	N/A	12.4	135
COVID-19 status				
Tested, positive	N/A	N/A	2.1	23
Tested, results pending	N/A	N/A	1.6	17
Not tested, presumed positive	N/A	N/A	5.0	55
Not tested, no perceived need for test	N/A	N/A	91.3	995
Frontline status				
Direct contact	N/A	N/A	51.9	566
Indirect/virtual contact	N/A	N/A	17.6	192
No contact	N/A	N/A	30.5	332
Specialty risk level				
Low	N/A	N/A	18.1	197
Moderate	N/A	N/A	32.8	357
High	N/A	N/A	44.1	481
Other	N/A	N/A	5.0	55
Profession				
Physician	N/A	N/A	31.2	340
Nurse	N/A	N/A	19.1	208
Physician, nursing, or medical assistant	N/A	N/A	6.1	67
Health technologist/technician	N/A	N/A	8.1	88
Medical trainee	N/A	N/A	17.5	191
Other clinical role	N/A	N/A	8.1	88
Other non-clinical role	N/A	N/A	9.9	108
**Interpersonal-level factors**				
Social support needs*	0.92	0.99	N/A	N/A
Reported change in living situation due to COVID-19	N/A	N/A	16.0	174
**Hospital-level factors**				
Reported change in role	N/A	N/A	73.9	806
Hours changed				
More hours	N/A	N/A	23.2	253
Fewer hours	N/A	N/A	30.7	335
No change	N/A	N/A	46.1	502
PPE shortage at beginning of pandemic				
Yes	N/A	N/A	48.3	526
No	N/A	N/A	42.8	467
Don’t know	N/A	N/A	8.9	97
Hospital policies				
Transparent*	4.30	0.84	N/A	N/A
Implemented quickly*	3.78	1.12	N/A	N/A
Difficult to follow*	2.34	1.05	N/A	N/A
Team cohesion*	21.30	4.26	N/A	N/A
**Community-level factors**				
HCW appreciation*	3.96	0.85	N/A	N/A
HCW stigmatization*	2.29	1.06	N/A	N/A
Satisfaction with state/local government response*	3.43	1.21	N/A	N/A
Satisfaction with federal government response*	1.83	1.10	N/A	N/A
Media consumption—hours per day	1.44	1.14	N/A	N/A

*Measured using 5-point Likert scale (5 = strongly agree; 1 = strongly disagree).

N/A = Not Applicable.

### Predictive analyses

**Fig 1:**
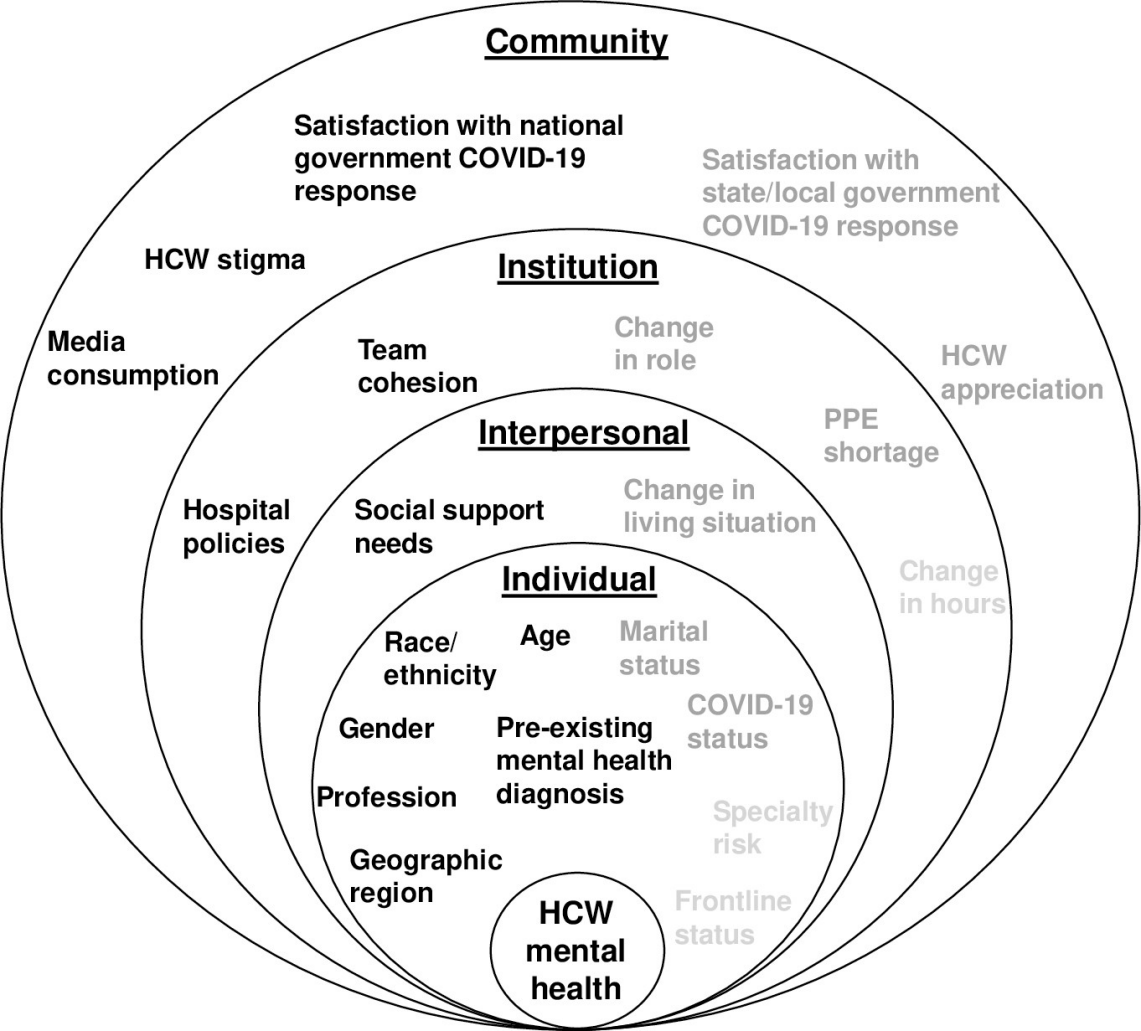
Socio-ecological model for mental health outcomes among healthcare workers during the COVID-19 pandemic. Socio-ecological factors in light grey text were not significantly associated with any mental health outcome in unadjusted and adjusted models. Socio-ecological factors in dark grey text were significant predictors of at least one mental health outcome in the unadjusted models. Socio-ecological factors in black text were significantly associated with at least one mental health outcome in the unadjusted and adjusted models.

The results of unadjusted and adjusted binary logistic regression analyses are summarized in Tables 2 and 3, respectively. Fig 1 depicts the socio-ecological model and specifies the degree to which each factor predicted mental health outcomes in unadjusted and adjusted models.

**Table 2:** Socio-ecological factors associated with mental health outcomes using unadjusted models.

	Probable MD		Probable GAD		Probable PTSD		Probable AUD	
* *	*OR (95% CI)*	*p-value*	*OR (95% CI)*	*p-value*	*OR (95% CI)*	*p-value*	*OR (95% CI)*	*p-value*
**Individual-level factors**								
Age (years)	0.98 (0.97–1.00)*	0.020	0.96 (0.94–0.98)***	<0.001	0.98 (0.97–0.99)**	0.004	0.99 (0.98–1.00)*	0.014
Gender								
Male	1.00 [Reference]	0.016	1.00 [Reference]	0.004	1.00 [Reference]	<0.001	1.00 [Reference]	0.007
Female	1.67 (1.10–2.58)*		1.82 (1.21–2.74)**		2.78 (1.90–4.08)***		1.45 (1.11–1.90)**	
Hispanic ethnicity								
Yes	1.56 (0.81–3.00)	0.187	1.67 (0.90–3.10)	0.107	1.99 (1.16–3.43)*	0.013	0.86 (0.51–1.46)	0.570
No	1.00 [Reference]		1.00 [Reference]		1.00 [Reference]		1.00 [Reference]	
Race								
White	1.00 [Reference]		1.00 [Reference]		1.00 [Reference]		1.00 [Reference]	
Black or African American	1.31 (0.62–2.75)	0.482	0.54 (0.21–1.39)	0.202	1.31 (0.71–2.44)	0.393	0.63 (0.35–1.12)	0.116
Asian	0.96 (0.57–1.61)	0.871	0.78 (0.47–1.30)	0.340	0.62 (0.39–0.99)*	0.045	0.44 (0.30–0.65)***	<0.001
Other	1.56 (0.58–4.23)	0.380	0.22 (0.03–1.62)	0.136	0.44 (0.13–1.50)	0.191	0.71 (0.31–1.63)	
Marital status								
Married	1.00 [Reference]		1.00 [Reference]		1.00 [Reference]		1.00 [Reference]	
Single	1.75 (1.21–2.52)**	0.003	1.67 (1.18–2.37)**	0.004	1.36 (1.00–1.86)*	0.049	1.25 (0.95–1.63)	0.108
Divorced or widowed	1.45 (0.75–2.80)	0.272	1.10 (0.56–2.17)	0.775	1.09 (0.62–1.93)	0.758	1.42 (0.88–2.29)	0.151
Pre-pandemic psychiatric illness								
Yes	3.19 (2.24–4.55)***	<0.001	3.26 (2.32–4.58)***	<0.001	2.62 (1.94–3.56)***	<0.001	1.41 (1.07–1.86)*	0.015
No	1.00 [Reference]		1.00 [Reference]		1.00 [Reference]		1.00 [Reference]	
Region								
Northeast	1.19 (0.77–1.84)	0.431	1.50 (0.98–2.32)	0.065	1.50 (1.04–2.16)*	0.029	1.34 (0.99–1.80)	0.056
South	1.33 (0.70–2.55)	0.385	1.49 (0.78–2.82)	0.226	1.37 (0.79–2.38)	1.367	1.27 (0.80–2.02)	0.307
Midwest	1.00 [Reference]		1.00 [Reference]		1.00 [Reference]		1.00 [Reference]	
West	1.34 (0.74–2.42)	0.340	1.63 (0.91–2.90)	0.100	1.29 (0.77–2.15)	0.336	1.24 (0.81–1.88)	0.326
COVID-19 status								
Tested, positive	1.32 (0.44–3.93)	0.620	1.55 (0.57–4.25)	0.391	1.57 (0.64–3.86)	0.327	0.71 (0.30–1.69)	0.438
Tested, results pending	0.39 (0.05–2.98)	0.365	0.75 (0.17–3.29)	0.698	1.96 (0.72–5.35)	0.191	1.50 (0.57–3.91)	0.411
Not tested, presumed positive	1.39 (0.69–2.83)	0.361	1.56 (0.80–3.03)	0.189	1.89 (1.06, 3.37)*	0.030	0.96 (0.55–1.66)	0.873
Not tested, no need for test	1.00 [Reference]		1.00 [Reference]		1.00 [Reference]		1.00 [Reference]	
Frontline status								
Direct	1.18 (0.79–1.78)	0.416	1.02 (0.70–1.49)	0.916	1.21 (0.87–1.67)	0.253	1.18 (0.90–1.55)	0.236
Indirect	1.52 (0.92–2.50)	0.103	1.33 (0.83–2.14)	0.233	0.91 (0.58–1.41)	0.665	0.76 (0.53–1.10)	0.146
None	1.00 [Reference]		1.00 [Reference]		1.00 [Reference]		1.00 [Reference]	
Specialty risk level								
Low	1.00 [Reference]		1.00 [Reference]		1.00 [Reference]		1.00 [Reference]	
Moderate	0.91 (0.58–1.43)	0.684	1.05 (0.68–1.62)	0.821	0.78 (0.53–1.14)	0.204	1.13 (0.82–1.57)	0.457
High	0.81 (0.52–1.25)	0.335	0.80 (0.52–1.21)	0.286	0.87 (0.61–1.24)	0.445	1.27 (0.93–1.73)	0.130
Profession								
Physician	1.00 [Reference]		1.00 [Reference]		1.00 [Reference]		1.00 [Reference]	
Nurse	2.74 (1.63–4.58)***	<0.001	1.63 (0.97–2.75)	0.068	2.19 (1.43–3.35)***	<0.001	1.02 (0.72–1.45)	0.901
Physician, nursing, or medical assistant	1.30 (0.54–3.11)	0.556	1.09 (0.46–2.57)	0.852	2.24 (1.22–4.12)**	0.009	1.06 (0.63–1.81)	0.818
Health technologist or technician	3.49 (1.87–6.52)***	<0.001	4.34 (2.44–7.71)***	<0.001	2.51 (1.46–4.31)**	0.001	0.80 (0.49–1.29)	0.358
Medical trainee	1.38 (0.76–2.50)	0.293	1.80 (1.07–3.05)*	0.028	1.41 (0.89–2.24)	0.148	1.00 (0.70–1.44)	0.979
Other clinical role	1.59 (0.76–3.34)	0.219	1.19 (0.56–2.53)	0.645	1.67 (0.93–2.98)	0.085	1.27 (0.80–2.04)	0.315
Other non-clinical role	3.02 (1.65–5.50)***	<0.001	3.58 (2.06–6.22)***	<0.001	2.83 (1.72–4.67)***	<0.001	1.39 (0.90–2.15)	0.134
**Interpersonal-level factors**								
Social support needs	2.64 (2.20–3.17)***	<0.001	2.75 (2.30–3.29)***	<0.001	2.22 (1.91–2.57)***	<0.001	1.21 (1.07–1.37)**	0.002
Change in living situation due to COVID-19								
Yes	1.57 (1.03–2.40)*	0.038	1.66 (1.11–2.49)*	0.014	1.56 (1.08–2.23)*	0.016	0.93 (0.67–1.29)	0.670
No	1.00 [Reference]		1.00 [Reference]		1.00 [Reference]		1.00 [Reference]	
**Hospital-level factors**								
PPE shortage—beginning of pandemic								
Yes	1.61 (1.11–2.34)*	0.013	2.00 (1.39–2.89)***	<0.001	1.64 (1.20–2.22)**	0.001	0.96 (0.75–1.24)	0.768
No	1.00 [Reference]		1.00 [Reference]		1.00 [Reference]		1.00 [Reference]	
Don’t know	1.77 (0.97–3.23)	0.062	2.36 (1.34–4.15)**	0.003	1.22 (0.72–2.09)	0.460	0.75 (0.48–1.18)	0.220
Roles changed								
Yes	1.26 (0.84–1.90)	0.265	1.78 (1.17–2.71)**	0.007	1.32 (0.94–1.85)	0.105	0.94 (0.71–1.23)	0.643
No	1.00 [Reference]		1.00 [Reference]		1.00 [Reference]		1.00 [Reference]	
Hours changed								
More hours	1.51 (0.99–2.29)	0.053	1.61 (1.08–2.40)*	0.019	1.37 (0.97–1.94)	0.078	0.78 (0.58–1.07)	0.122
Fewer hours	1.05 (0.70–1.59)	0.804	1.15 (0.77–1.70)	0.496	1.00 (0.71–1.40)	0.994	1.05 (0.80–1.39)	0.721
No change	1.00 [Reference]		1.00 [Reference]		1.00 [Reference]		1.00 [Reference]	
** Hospital policies**								
Transparent	0.57 (0.48–0.68)***	<0.001	0.59 (0.50–0.70)***	<0.001	0.59 (0.50–0.69)***	<0.001	0.96 (0.84–1.11)	0.613
Implemented quickly	0.67 (0.58–0.78)***	<0.001	0.67 (0.58–0.77)***	<0.001	0.70 (0.62–0.79)***	<0.001	0.94 (0.85–1.05)	0.258
Difficult to follow	1.60 (1.37–1.87)***	<0.001	1.40 (1.20–1.62)***	<0.001	1.31 (1.15–1.49)***	<0.001	0.95 (0.85–1.07)	0.421
Team cohesion	0.87 (0.83–0.90)***	<0.001	0.87 (0.84–0.91)***	<0.001	0.88 (0.85–0.91)***	<0.001	0.99 (0.96–1.02)	0.528
**Community-level factors**								
HCW appreciation	0.67 (0.56–0.81)***	<0.001	0.73 (0.61–0.88)**	0.001	0.71 (0.61–0.84)***	<0.001	0.94 (0.81–1.08)	0.362
HCW stigmatization	1.49 (1.28–1.74)***	<0.001	1.39 (1.19–1.61)***	<0.001	1.71 (1.50–1.95)***	<0.001	1.16 (1.04–1.30)**	0.009
Satisfaction with state/local government response	0.79 (0.69–0.91)**	0.001	0.81 (0.71–0.92)**	0.002	0.84 (0.75–0.94)**	0.002	1.01 (0.92–1.12)	0.843
Satisfaction with federal government Response	0.89 (0.76–1.05)	0.175	0.80 (0.68–0.95)*	0.010	0.90 (0.79–1.03)	0.139	0.91 (0.81–1.02)	0.091
Media consumption (hours per day)	1.17 (1.03–1.33)*	0.016	1.23 (1.09–1.39)**	0.001	1.19 (1.06–1.34)**	0.003	1.06 (0.96–1.18)	0.257

OR = Odds Ratio.

CI = Confidence Interval.

*0.05 > p-value > = 0.01.

**0.01 > p-value > = 0.001.

***p-value < 0.001.

**Table 3:** Socio-ecological factors associated with mental health outcomes using multivariable models.

	Probable MD		Probable GAD		Probable PTSD		Probable AUD	
* *	*adjOR (95% CI)*	*p-value*	*adjOR (95% CI)*	*p-value*	*adjOR (95% CI)*	*p-value*	*adjOR (95% CI)*	*p-value*
**Individual-level factors**								
Age (years)	1.01 (0.98–1.03)	0.584	0.97 (0.95–0.99)*	0.012	1.00 (0.98–1.02)	0.723	0.98 (0.97–1.00)*	0.015
Gender								
Male	1.00 [Reference]	0.886	1.00 [Reference]	0.542	1.00 [Reference]	0.002	1.00 [Reference]	0.076
Female	0.96 (0.57–1.63)		1.18 (0.70–2.00)		2.05 (1.30–3.23)**		1.32 (0.97–1.80)	
Hispanic ethnicity								
Yes	1.12 (0.48–2.61)	0.794	0.95 (0.40–2.22)	0.897	1.55 (0.78–3.07)	0.213	0.61 (0.34–1.07)	0.085
No	1.00 [Reference]		1.00 [Reference]		1.00 [Reference]		1.00 [Reference]	
Race								
White	1.00 [Reference]		1.00 [Reference]		1.00 [Reference]		1.00 [Reference]	
Black or African American	0.92 (0.35–2.37)	0.858	0.23 (0.07–0.74)*	0.014	1.12 (0.52–2.42)	0.770	0.50 (0.26–0.94)*	0.033
Asian	1.26 (0.66–2.40)	0.484	0.78 (0.41–1.50)	0.458	0.68 (0.39–1.20)	0.179	0.36 (0.23–0.55)***	<0.001
Other	2.75 (0.86–8.78)	0.088	0.19 (0.02–1.87)	0.156	0.49 (0.12–1.96)	0.314	0.69 (0.29–1.63)	0.397
Marital status								
Married	1.00 [Reference]		1.00 [Reference]		1.00 [Reference]		1.00 [Reference]	
Single	1.10 (0.67–1.79)	0.704	0.94 (0.58–1.53)	0.803	0.83 (0.55–1.24)	0.356	1.12 (0.82–1.54)	0.488
Divorced or widowed	1.23 (0.58–2.61)	0.590	1.17 (0.52–2.64)	0.704	0.79 (0.40–1.54)	0.484	1.50 (0.89–2.52)	0.130
Pre-pandemic psychiatric illness								
Yes	2.49 (1.63–3.79)***	<0.001	2.30 (1.52–3.50)***	<0.001	1.88 (1.31–2.69)**	0.001	1.26 (0.93–1.70)	0.140
No	1.00 [Reference]		1.00 [Reference]		1.00 [Reference]		1.00 [Reference]	
Region								
Northeast	1.21 (0.79–2.09)	0.499	1.78 (1.01–3.14)*	0.046	1.54 (0.98–2.42)	0.064	1.36 (0.97–1.90)	0.072
South	1.65 (0.72–3.78)	0.234	2.91 (1.25–6.80)*	0.013	1.58 (0.79–3.16)	0.194	1.71 (1.02–2.87)*	0.043
Midwest	1.00 [Reference]		1.00 [Reference]		1.00 [Reference]		1.00 [Reference]	
West	1.23 (0.58–2.57)	0.592	1.36 (0.65–2.88)	0.417	1.20 (0.64–2.26)	0.564	1.40 (0.88–2.24)	0.161
COVID-19 status								
Tested, positive	1.22 (0.35–4.23)	0.751	1.23 (0.38–4.00)	0.729	1.63 (0.56–4.75)	0.367	0.74 (0.29–1.85)	0.515
Tested, results pending	0.49 (0.06–4.24)	0.516	1.65 (0.30–9.12)	0.566	2.69 (0.85–8.51)	0.093	1.94 (0.71–5.30)	0.197
Not tested, presumed positive	0.97 (0.42–2.25)	0.935	1.25 (0.55–2.82)	0.599	1.46 (0.73–2.92)	0.290	0.89 (0.49–1.60)	0.687
Not tested, no need for test	1.00 [Reference]		1.00 [Reference]		1.00 [Reference]		1.00 [Reference]	
Frontline status								
Direct	1.07 (0.59–1.96)	0.815	0.85 (0.47–1.52)	0.579	1.22 (0.75–1.98)	0.433	1.24 (0.86–1.80)	0.246
Indirect	1.30 (0.70–2.42)	0.411	0.99 (0.53–1.83)	0.968	0.82 (0.48–1.42)	0.481	0.85 (0.57–1.29)	0.450
None	1.00 [Reference]		1.00 [Reference]		1.00 [Reference]		1.00 [Reference]	
Specialty risk level								
Low	1.00 [Reference]		1.00 [Reference]		1.00 [Reference]		1.00 [Reference]	
Moderate	1.10 (0.61–1.99)	.760	1.18 (0.66–2.11)	0.572	0.73 (0.44–1.19)	0.207	1.15 (0.78–1.70)	0.469
High	0.91 (0.48–1.75)	0.781	0.87 (0.45–1.66)	0.666	0.77 (0.45–1.31)	0.337	1.11 (0.73–1.69)	0.631
Profession								
Physician	1.00 [Reference]		1.00 [Reference]		1.00 [Reference]		1.00 [Reference]	
Nurse	2.32 (1.21–4.48)*	0.012	1.17 (0.60–2.31)	0.644	1.25 (0.73–2.12)	0.418	0.70 (0.46–1.05)	0.085
Physician, nursing, or medical assistant	0.87 (0.28–2.67)	0.810	0.42 (0.13–1.33)	0.140	1.34 (0.62–2.10)	0.463	0.73 (0.40–1.32)	0.297
Health technologist or technician	2.66 (1.16–6.12)*	0.021	3.70 (1.67–8.23)**	0.001	1.25 (0.61–2.53)	0.541	0.62 (0.35–1.11)	0.109
Medical trainee	1.04 (0.50–2.18)	0.919	1.09 (0.55–2.17)	0.797	1.03 (0.57–1.85)	0.925	0.68 (0.44–1.06)	0.086
Other clinical role	1.22 (0.48–3.09)	0.674	0.71 (0.27–1.84)	0.475	1.13 (0.54–2.34)	0.746	1.22 (0.71–2.11)	0.472
Other non-clinical role	2.05 (0.91–4.66)	0.085	2.48 (1.12–5.50)*	0.025	2.06 (1.07–3.98)*	0.031	1.28 (0.76–2.15)	0.357
**Interpersonal-level factors**								
Social support needs	2.22 (1.78–2.78)***	<0.001	2.30 (1.84–2.88)***	<0.001	1.76 (1.47–2.10)***	<0.001	1.17 (1.01–1.35)*	0.038
Change in living situation due to COVID-19								
Yes	1.12 (0.66–1.89)	0.683	1.23 (0.73–2.06)	0.435	1.32 (0.84–2.05)	0.226	0.91 (0.64–1.30)	0.596
No	1.00 [Reference]		1.00 [Reference]		1.00 [Reference]		1.00 [Reference]	
**Hospital-level factors**								
PPE shortage—beginning of pandemic								
Yes	0.86 (0.54–1.36)	0.514	1.11 (0.70–1.76)	0.666	1.03 (0.71–1.49)	0.887	0.82 (0.62–1.09)	0.172
No	1.00 [Reference]		1.00 [Reference]		1.00 [Reference]		1.00 [Reference]	
Don’t know	1.25 (0.61–2.56)	0.545	2.02 (1.00–4.08)	0.051	0.96 (0.51–1.81)	0.891	0.74 (0.45–1.22)	0.237
Roles changed								
Yes	0.93 (0.56–1.53)	0.764	1.40 (0.83–2.37)	0.210	1.05 (0.70–1.59)	0.808	0.95 (0.70–1.29)	0.751
No	1.00 [Reference]		1.00 [Reference]		1.00 [Reference]		1.00 [Reference]	
Hours changed								
More hours	1.18 (0.70–1.97)	0.539	1.11 (0.67–1.85)	0.682	1.14 (0.75–1.76)	0.539	0.75 (0.53–1.05)	0.095
Fewer hours	1.24 (0.74–2.07)	0.420	1.01 (0.61–1.69)	0.958	1.06 (0.69–1.61)	0.803	1.19 (0.87–1.64)	0.275
No change	1.00 [Reference]		1.00 [Reference]		1.00 [Reference]		1.00 [Reference]	
** Hospital policies**								
Transparent	0.93 (0.71–1.24)	0.627	0.98 (0.73–1.30)	0.860	0.85 (0.67–1.09)	0.194	1.00 (0.82–1.22)	0.994
Implemented quickly	0.94 (0.76–1.16)	0.536	0.93 (0.75–1.14)	0.477	1.01 (0.85–1.21)	0.918	0.97 (0.84–1.12)	0.706
Difficult to follow	1.35 (1.10–1.66)**	0.004	1.06 (0.85–1.31)	0.612	1.01 (0.85–1.21)	0.903	0.91 (0.80–1.05)	0.192
Team cohesion	0.98 (0.93–1.03)	0.438	0.95 (0.90–1.00)	0.069	0.95 (0.90–0.99)*	0.022	1.01 (0.97–1.05)	0.748
**Community-level factors**								
HCW appreciation	0.84 (0.66–1.06)	0.142	0.97 (0.77–1.24)	0.820	0.98 (0.80–1.20)	0.838	1.15 (1.01–1.31)	0.720
HCW stigmatization	1.13 (0.94–1.38)	0.203	1.03 (0.85–1.25)	0.756	1.42 (1.21–1.66)***	<0.001	1.15 (1.01–1.31)*	0.038
Satisfaction with state/local government response	0.93 (0.79–1.11)	0.429	0.97 (0.82–1.15)	0.733	1.00 (0.86–1.15)	0.942	1.05 (0.94–1.18)	0.409
Satisfaction with federal government response	0.90 (0.73–1.10)	0.306	0.77 (0.62–0.95)*	0.014	0.89 (0.75–1.06)	0.187	0.89 (0.78–1.01)	0.072
Media consumption (hours per day)	1.16 (0.98–1.37)	0.078	1.37 (1.18–1.60)***	<0.001	1.22 (1.06–1.41)**	0.005	1.10 (0.98–1.23)	0.113

adjOR = adjusted Odds Ratio.

CI = Confidence Interval.

*0.05 > p-value > = 0.01.

**0.01 > p-value > = 0.001.

***p-value < 0.001.

#### Probable major depression

In unadjusted models, several factors were significantly associated with the odds of probable MD. Nagelkerke’s *R*^2^ for the full adjusted model predicting probable MD was 0.31. Two individual-, one interpersonal-, and one institutional-level factors retained statistical significance. Specifically, participants with a pre-pandemic mental health diagnosis had 2.49 odds (95% confidence interval [CI]: 1.63–3.79; p<0.001) of probable MD. Nurses and health technicians/technologists had 2.32 (95% CI: 1.21–4.48; p = 0.012) and 2.66 (95% CI: 1.16–6.12; p = 0.021) odds, respectively, of probable MD, relative to physicians. Each unit increase on the item assessing social support needs was associated with 2.22 (95% CI: 1.78–2.78; p<0.001) odds, and each unit increase on the item assessing perceived difficulty adhering to hospital COVID-19 policies was associated with 1.35 (95% CI: 1.10–1.66; p = 0.004) odds of probable MD.

#### Probable generalized anxiety disorder

In the unadjusted models, several factors were associated with the odds of probable GAD. Nagelkerke’s *R*^2^ for the multivariable model predicting probable GAD was 0.37. Five individual-, one interpersonal-, and two community-level factors retained statistical significance. At the individual-level, each one-year increase in age was associated with 0.97 odds (95% CI: 0.95–0.99; p = 0.012) of probable GAD. Participants identifying as African American, compared to white, had a 0.23 (95% CI: 0.07–0.74; p = 0.014) odds of probable GAD. Participants with a pre-pandemic mental illness had 2.30 odds (95% CI: 1.52–3.50; p<0.001) of probable GAD. Participants from the Northeast (adjusted OR [adjOR] = 1.78; 95% CI: 1.01–3.14; p = 0.046) and South (adjOR = 2.91; 95% CI: 1.25–6.80; p = 0.013) regions were at increased odds of probable GAD, compared to those living in the Midwest. Health technologists/technicians (adjOR = 4.34; 95% CI: 1.67–8.23; p = 0.001) and those working in other non-clinical roles (adjOR = 2.48; 95% CI: 1.12–5.50; p = 0.025) were at increased odds of probable GAD compared to physicians. At the interpersonal level, each unit increase in social support needs was associated with 2.30 increased odds of probable GAD (95% CI: 1.84–2.88; p<0.001). At the community level, every one-hour increase in media consumption was associated with 1.37 odds (95% CI: 1.18–1.60; p<0.001) of probable GAD. Each unit increase on the Likert scale assessing satisfaction with the federal government response reduced the odds (adjOR = 0.77; 95% CI: 0.62–0.95; p = 0.014) of probable GAD.

#### Probable posttraumatic stress disorder

In unadjusted analyses, several factors were associated with the odds of probable PTSD. Nagelkerke’s *R*^2^ for the adjusted model predicting probable PTSD was 0.30. Three individual-, one interpersonal-, one institutional-, and two community-level factors retained statistical significance. At the individual-level, female gender was associated with 2.05 odds of probable PTSD (95% CI: 1.30–3.23; p = 0.002) compared to male gender. Reporting a pre-pandemic psychiatric illness was associated with 1.88 odds of probable PTSD (95% CI: 1.31–2.69; p = 0.001). Working in a non-clinical role (vs. being a physician) was associated with 2.06 odds of probable PTSD (95% CI: 1.07–3.98; p = 0.031). At the interpersonal level, each unit increase social support needs was associated with 1.76 times the odds of probable PTSD (95% CI: 1.47–2.10; p<0.001). At the institutional level, higher perceived team cohesion was protective against probable PTSD (adjOR = 0.95; 95% CI: 0.90–0.99; p = 0.022). For the community level, each unit increase in the item assessing perceived HCW stigmatization was associated with 1.42 odds of probable PTSD (95% CI: 1.21–1.66; p<0.001). Every one-hour increase in daily media consumption was associated with 1.22 odds of probable PTSD (95% CI: 1.06–1.41; p = 0.005).

#### Probable alcohol use disorder

In the unadjusted analyses, several factors were associated with the odds of probable AUD. Nagelkerke’s *R*^2^ for the multivariable model predicting probable AUD was 0.12. Three individual-, one interpersonal-, and one community-level factors retained statistical significance. At the individual-level, each one-year increase in age was associated with 0.98 odds (95% CI: 0.97–1.00; p = 0.015) of probable AUD. African American (adjOR = 0.50; 95% CI: 0.26–0.94; p = 0.033) and Asian participants (adjOR = 0.36; 95% CI: 0.23–0.55; p<0.001) were at reduced odds of probable AUD compared to white participants. Participants from the South were at 1.71 odds of probable AUD, relative to those from the Midwest (95% CI: 1.02–2.87; p = 0.043). At the interpersonal level, those in need of additional social support had 1.17 odds of probable AUD (95% CI: 1.01–1.35; p = 0.038). At the community-level, perceived HCW stigmatization was associated with 1.15 odds of probable AUD (95% CI: 1.01–1.31; p = 0.038).

## Discussion

This cross-sectional study including 1,092 HCWs across the US in May 2020 identified the prevalence of probable MD, PTSD, GAD, and AUD as 13.9%, 22.8%, 15.6%, and 42.8%, respectively. In multivariable models that adjusted for factors from four socio-ecological levels, the only consistently significant predictor of all outcomes was one interpersonal level-factor–greater social support needs–while the other factors varied by mental health outcome.

The prevalence of adverse mental health outcomes in our sample were similar to those of another survey study including 5,550 HCWs affiliated with a major academic center in Missouri [11]. The authors found that the prevalence of moderate to high levels of depression and anxiety in their sample were 15.9% and 13.0%, respectively. However, a study of HCWs in NYC (n = 657) estimated greater burdens of adverse mental health outcomes, with 48%, 33%, and 57% of their sample having probable MD, GAD, and PTSD, respectively [4]. This discrepancy could be due to the unique location and high burden of COVID-19 cases in NYC compared with other geographical areas in the US. Additional studies are needed to validate the burden of mental health outcomes among HCWs in the US.

Notably, 42.8% of our sample met our criterion for probable AUD. One survey of physicians in Poland found that over half of their participants increased their alcohol consumption during the COVID-19 pandemic and just under 20% drank more than seven drinks in one occasion [32]. Additional studies are warranted to further explore changes in alcohol consumption among HCWs and provide appropriate interventions.

Previous COVID-19 studies identified similar individual-level risk factors for mental health outcomes, including younger age, female gender, and having a pre-existing mental health condition [2, 10, 11, 33–35]. For example, in a study conducted in China, female gender was associated with 1.94 (95%CI: 1.26–2.98), 1.69 (95%CI: 1.23–2.33), and 1.45 (95%CI: 1.09–1.96) times the odds of severe depression, anxiety, and PTSD, respectively, after controlling for age, marital status, education level, occupation, geographic region, frontline status, and type of hospital [8]. Also similar to our findings, other studies identified that mental health outcomes were worse for nurses compared with doctors [2] and nonmedical HCWs compared with medical HCWs [36]. Perhaps residual confounding from higher socioeconomic status and power of physicians compared to nursing and nonmedical staff could explain these differences. Notably, physicians and nurses comprised the majority of the sample in our study and other HCW studies [2]. Additional studies are needed to better understand the impact of the pandemic on a wider range of HCWs, including custodial, housekeeping, transportation, and food services staff.

We also explored whether mental health outcomes varied by race/ethnic group. Many have postulated that African American/Black, Latinx, and Indigenous HCWs bear the weight of the COVID-19 pandemic, as most patients are from underrepresented racial/ethnic groups and HCWs of color have disproportionately contracted COVID-19 compared with their white colleagues [37, 38]. Yet, our findings suggest that African American/Black respondents had decreased risk of GAD and AUD compared with white respondents. The survey study of HCWs in Missouri also found that underrepresented participants, which included those identifying as African American/Black, Hispanic, Hawaiian/Pacific Islander, and Native American, had lower levels of stress and depression and higher levels of wellbeing compared with white participants [11]. However, only 12.7% and 10.4% of our sample and the Missouri sample, respectively, included underrepresented groups, when 30.3% of HCWs in the US identify as African American/Black, Hispanic, Hawaiian/Pacific Islander, or Native American [39]. Furthermore, both studies were conducted before the Black Lives Matter Movement that followed the publicized police killings of unarmed Black men and women during the COVID-19 pandemic, including George Floyd and Breonna Taylor, which likely impacts Black HCWs’ mental health [40, 41]. Thus, studies are needed that over-sample from racial/ethnic groups that are underrepresented in medicine to understand the ways in which COVID-19 stressors interact with experienced racism within and outside the hospital.

Needing more social support was the only factor associated with increased risk of all four mental health outcomes in multivariable models. Similarly, studies conducted among HCWs in China during the COVID-19 pandemic found that social support was protective for mental health [9, 12, 13]. From our qualitative findings gathered within the same survey, many respondents suggested that their most upsetting experience was being isolated from their friends and families [15]. Thus, providing innovative avenues for HCWs to receive social support while maintaining physical distance is critical to promote their resilience.

Furthermore, we identified team cohesion as an institutional-level factor associated with mental health outcomes. Studies from the Middle East Respiratory Syndrome (MERS) pandemic [23, 42, 43] and our qualitative paper on HCWs’ experiences [15] also found that higher levels of team cohesion and supervisor support were associated with increased morale among HCWs. Difficulty adhering to hospital policies was another institutional-level factor associated with increased risk of probable MD in our multivariable model. In fact, some respondents from our qualitative study communicated that their most upsetting experience was physically providing clinical care to patients while wearing extensive PPE [15]. Many HCWs expressed that the protective gear made it difficult to provide emotional support to patients, especially because they could not see their faces with masks and face shields. Thus, interventions that aim to improve cohesion within hospital teams and innovate strategies that mitigate PPE clinical care barriers could help support HCWs within the hospital.

Lastly, our study found significant community-level factors associated with HCW mental health outcomes. Low satisfaction with the national US government’s COVID-19 response was associated with increased risk of probable GAD and is consistent with our qualitative findings [15]. We also identified perceived stigma against HCWs as a community-level factor associated with increased risk of adverse mental health outcomes, which is consistent with a study conducted in China [9]. Our analysis also complements studies from the MERS outbreak that identified media exposure as a risk factor for adverse mental health outcomes [23, 44], highlighting the importance of encouraging HCWs to limit pandemic-related media usage.

Our study has some notable strengths and limitations. First, our large sample included over 1,000 HCWs during the peak of the first wave of the COVID-19 outbreak in the US. However, this is a non-representative convenience sample, which threatens generalizability, particularly the prevalence estimates identified in this study. We purposefully developed our survey by using a comprehensive list of validated measures of mental health outcomes and individual-, interpersonal-, institutional-, and community-level socio-ecological factors based on findings from previous pandemics. However, our qualitative analysis of HCWs’ most upsetting and hopeful experiences from the same survey roll-out indicates that we omitted key socio-ecological factors that are likely to influence mental health, such as childcare support [15]. This limitation reflects another important strength of our study, which is that the mixed methods approach of this survey allowed us to contextualize and triangulate quantitative findings with our qualitative analysis of HCW’s most upsetting and hopeful experiences. The cross-sectional nature of the data also limits our ability to assess whether there might be causal relationship(s) between socio-ecological factors and mental health outcomes, and in what direction. Although we were not able to assess response rate and bias due to the sampling methods and anonymous nature of the survey, we believe that the anonymity enabled HCWs to respond openly and honestly to the questions provided. Our sample was 72% female, which is similar to the gender distribution within the healthcare workforce in the US (i.e. 76% female) [45]. However, the majority of our respondents were white HCWs; additional studies should survey more representative samples of HCWs, including increased racial/ethnic diversity, to understand the experiences and mental health outcomes of all HCWs. The distribution of our sample based on geographic region includes mostly HCWs from the Northeast, with only 9.4% of our sample working in the South. Based on data from the Bureau of Labor Statistics, 24.1% of HCWs in the US work in the Northeast while 33.5% work in the South [46]. As different areas may face different burdens of COVID-19 and socio-ecological risk factors, additional studies are needed to understand how HCWs in different regions are differentially impacted by COVID-19.

## Conclusion

In sum, we conducted a cross-sectional survey study to assess socio-ecological factors associated with probable MD, GAD, PTSD, and AUD among 1,092 HCWs during the COVID-19 pandemic in the US. As social support needs were predictive of all four mental health outcomes and team cohesion was predictive for probable PTSD, there is a need to create interventions that strengthen social networks within and outside the hospital while adhering to physical distancing guidelines. For example, a virtual peer support group including frontline HCWs and licensed mental health providers was rolled out in Wuhan, China that provided HCWs with social support, active listening, sleep hygiene and mindfulness practices, and problem-solving [47]. Another approach for intervention development could involve targeting multiple socio-ecological levels based on the risk factors we identified. For example, an academic medical center in Connecticut rolled out a tiered approach to support HCWs by providing services at three socio-ecological levels simultaneously: individual, team, and community [48]. The individual-level included wellness checks, team-level included a buddy/peer support system, and community-level included stress and resilience town halls. Rolling out and scaling up similar interventions across the US could equip HCWs with resilience-building psychological and social support tools. We hope that our findings will be used to inform strategies to better care for our caregivers working on the frontlines of this and future pandemics.

## Supporting information

S1 Checklist STROBE statement—checklist of items that should be included in reports of cross-sectional studies. (DOCX)

S1 File (PDF)
